# Estimation of Fish Biomass Using Environmental DNA

**DOI:** 10.1371/journal.pone.0035868

**Published:** 2012-04-26

**Authors:** Teruhiko Takahara, Toshifumi Minamoto, Hiroki Yamanaka, Hideyuki Doi, Zen'ichiro Kawabata

**Affiliations:** 1 Research Institute for Humanity and Nature, Kyoto, Japan; 2 Institute for Sustainable Sciences and Development, Hiroshima University, Higashi-Hiroshima, Japan; 3 Department of Environmental Solution Technology, Faculty of Science and Technology, Ryukoku University, Otsu, Shiga, Japan; Argonne National Laboratory, United States of America

## Abstract

Environmental DNA (eDNA) from aquatic vertebrates has recently been used to estimate the presence of a species. We hypothesized that fish release DNA into the water at a rate commensurate with their biomass. Thus, the concentration of eDNA of a target species may be used to estimate the species biomass. We developed an eDNA method to estimate the biomass of common carp (*Cyprinus carpio* L.) using laboratory and field experiments. In the aquarium, the concentration of eDNA changed initially, but reached an equilibrium after 6 days. Temperature had no effect on eDNA concentrations in aquaria. The concentration of eDNA was positively correlated with carp biomass in both aquaria and experimental ponds. We used this method to estimate the biomass and distribution of carp in a natural freshwater lagoon. We demonstrated that the distribution of carp eDNA concentration was explained by water temperature. Our results suggest that biomass data estimated from eDNA concentration reflects the potential distribution of common carp in the natural environment. Measuring eDNA concentration offers a non-invasive, simple, and rapid method for estimating biomass. This method could inform management plans for the conservation of ecosystems.

## Introduction

Information about the distribution of a species is a critical component of understanding their ecology and extinction risks and is important for conservation of populations [1]. However, precise estimates of distribution are often hindered by the complex microhabitat topology and vegetation, particularly in aquatic systems. Recently, environmental DNA (hereafter, eDNA) has been used to document the distributions of aquatic vertebrate species [2]–[6]. Detection of short, species-specific DNA fragments in the water may increase the accuracy and decrease the cost of surveys and allow detection of rare or invasive species [2]. For example, researchers have used eDNA to document the presences of bullfrog tadpoles [3], silver and bighead carp [4], and frogs and salamanders [5] in a range of water bodies.

Absence/presence data can be used to illustrate species distribution, and eDNA has often been used to document presence/absence of aquatic species. However, knowledge of species biomass is critical to estimate the production and material cycling of ecosystems [1]. Biomass is a fundamental biological parameter, but it is often difficult to accurately estimate, particularly for aquatic organisms such as fish. Biomass information plays a major role in conserving rare and endangered species and in managing population sizes [1]. The fecal DNA of terrestrial vertebrates at the soil surface is known to reflect the relative biomass [7]. The eDNA technique has been applied to monitor virus concentrations in a lake [8]. Assuming that aquatic vertebrates release eDNA into the water (from feces, secretions, or tissues) in proportion with their biomass, eDNA could be used to estimate species biomass by measuring the number of eDNA copies in a sample of water.

Our objective was to develop a non-invasive eDNA-based method for estimating the biomass of fish using the common carp, *Cyprinus carpio* L., as a model organism. We conducted a series of laboratory experiments, pond experiments, and a field survey in a freshwater lagoon. We first evaluated the effects of time and temperature on eDNA concentrations in the laboratory. Using these data, we developed a model to estimate the carp biomass based on the number of eDNA copies. The concentration of eDNA is a function of the rate of release and the rate of breakdown, both of which are affected by ambient temperature. For example, Dejean et al. [9] reported that eDNA of fish (target length = 98 bp) or anuran tadpoles (79 bp) was detectable for 25 days after the removal of individuals at 8–11°C or for 21 days at 17±1°C. Also, dissolved DNA fragments (ca. 400 bp) could be detected in water up to one week at 18°C [10]. However, the effects of different temperature conditions on eDNA concentrations have not been clarified. Using outdoor artificial ponds, we optimized a method to evaluate the concentration of eDNA from a large volume of water and evaluated the relationship between eDNA concentrations and carp biomass/abundance. Last, we tested whether this method could estimate the biomass of a natural carp population in a lagoon system and evaluated the effects of the environmental factors (e.g., water temperature and habitat type) on the distribution of the carp eDNA.

## Materials and Methods

### Study species

We used common carp *Cyprinus carpio* L. for this study. This species is an ideal model organism for a number of reasons. First, carp are one of the most widely transported species in the world, being used as ornamental fish and for sport fishing and human consumption [11]. Second, introductions of common carp often cause ecological disruption at both community and ecosystem levels [12], [13]. For example, common carp can dramatically alter water quality, macrophyte abundance and composition, and invertebrate richness and diversity [14], [15]. Last, the impact of common carp is dependent on the relative densities of its populations [16].

### Real-time quantitative PCR

The quantification of eDNA was performed using real-time TaqMan® PCR with a StepOne-Plus™ Real-Time PCR system (Applied Biosystems, Foster City, CA, USA). The mitochondrial cytochrome *b* gene fragments were amplified and quantified with primers CpCyB_496F (5′-GGTGGGTTCTCAGTAGACAATGC-3′), CpCyB_573R (5′-GGCGGCAATAACAAATGGTAGT-3′), and CpCyB_550p probe (5′-FAM-CACTAACACGATTCTTCGCATTCCACTTCC-TAMRA-3′). These primers are specific to common carp and amplify a 78 bp fragment of the cytochrome *b* gene. The specificity of the primers was tested with the sequences of 56 species (all species for which the sequence data of the target region were available), representing 19 families that inhabit Lake Biwa, Japan, the field site. The primers were designed to have at least three mismatches with non-target species; the multiple alignment is shown in Table S1. In Japan, Japanese indigenous-type carp coexist with a morphologically-similar domesticated carp that belongs to the same genetic cluster as the carp found throughout the Eurasian continent [17]. The ecological differences between the two types have not been clarified in the natural environment. In this study we therefore designed the primers and the probe so that the both types of carps could be detected.

Each TaqMan reaction contained 900 nM of each primer and 125 nM TaqMan probe in 1× PCR master mix (TaqMan gene expression master mix; Applied Biosystems) and 5 µL of the DNA solution (2 µL during analysis of the aquarium experiments). The total volume of each reaction mixture was 20 µL. The PCR conditions were as follows: 2 min at 50°C, 10 min at 95°C, and 40 cycles of 15 s at 95°C and 60 s at 60°C. Quantitative real-time PCR (qPCR) was performed in triplicate, and the mean value was using during assays. Because one copy of the DNA was detected in at least two wells in each triplicate, we defined the limit of detection (LOD) for carp DNA using qPCR assay as one copy. If any of the triplicates showed a negative result, it was assigned a value of zero. PCR products of the target sequences were cloned into pGEM-T Easy Vector (Promega, Tokyo, Japan), and a dilution series of the plasmid containing 3×10^1^ to 3×10^4^ copies were amplified as standards in triplicate in all qPCR assays. Three wells of no-template negative control were adopted in all qPCR assays and showed no amplification.

The *in silico* specificity screen was performed using Primer-BLAST (http://www.ncbi.nlm.nih.gov/tools/primer-blast/). The result showed that two species in Lake Biwa (*Mylopharyngodon piceus* and *Opsariichthys uncirostris uncirostris*) were potentially amplified with our primer set. *Mylopharyngodon piceus* appears not to be able to reproduce in Lake Biwa, and this species was only recorded in surveys in 1953 [Shiga Prefectural Fisheries Experimental Station, http://www.geocities.co.jp/NatureLand/2851/txt0101003.html (in Japanese)]. The probe designed in this study had four mismatches to the corresponding sequence of *O. uncirostris uncirostris*. Furthermore, direct sequencing of qPCR amplicons showed that amplified fragments were certainly derived from carp DNA (see Results). Moreover, we used these primers to try to amplify tissue-extracted DNA of closely related and non-targeted species (*Carassius auratus langsdorfii*, *Carassius cuvieri*, and *Carassius buergeri grandoculis*). These tests resulted in no amplification or amplification below the LOD. Thus, our experimental system rarely overestimated the carp eDNA concentration.

### Experiment 1: aquarium experiments

Juvenile common carp were hatched and reared at Tamaki Station at the National Research Institute of Aquaculture (NRIA), Fisheries Research Agency, Mie Prefecture, Japan. The fish were then transported to a laboratory at the Research Institute for Humanity and Nature (RIHN), Kyoto Prefecture. The carp were held in separate 66 L plastic holding containers (∼25 individuals per container) in which the water was continuously filtered. The fish were fed a commercial diet (Saki-Hikari®, Kyorin Co. Ltd., Hyogo, Japan) three times a week, and were held at 19±1°C under a 12 h∶12 h light-dark cycle. All procedures were conducted in accordance with the current laws in Japan on experimental animals and were approved by the safety management committee for experiments of the RIHN.

We evaluated the relationships between eDNA concentrations in the aquaria and three factors (duration, water temperature, and fish biomass). We conducted three aquarium experiments using plastic tanks (30×45×25 cm) filled with 9 L of aged-tap water. The water in the tanks was continuously aerated through a filter. In each experiment, carp were randomly assigned from the holding container to the tanks, and were fed a diet that did not contain any common carp materials (confirmed with qPCR assay) every three days.

1. To evaluate time-dependent changes in eDNA concentrations, we quantified the number of eDNA copies in the water 1, 2, 3, 6, and 9 d after the carp were introduced to the tank. The tanks were stocked with one or three fish per tank, and each density was triplicated. The water in the tanks was maintained at 19±1°C. We collected a 20-mL water sample from each tank at each time point. We measured the wet-weight of each fish at the end of the experiment (15.0±3.5 g, n = 12, mean ±1 SD). Immediately after collection, the water was filtered using a centrifugal filter unit (Amicon Ultra-15, 30-kDa cutoff, UFC903096; Millipore, Billerica, MA, USA). The sample solution was then concentrated to a volume of 200 µL and stored in 1.5-mL microtube (Eppendorf®) at −25°C. The eDNA from each sample solution was extracted using a DNeasy blood and tissue kit (Qiagen, Hilden, Germany) in a final volume of 100 µL. To confirm the absence of the carp eDNA in the water prior to the experiments, two tanks without carp were prepared. A water sample was collected on day 6 and treated as described above.
2. To evaluate the effect of temperature, we held fish (n = 3 fish tank^−1^) at 7, 15, or 25°C (±1°C) (n = 4 tanks per treatment group). We controlled the water temperature in each tank using a heater with a built-in thermostat. We collected a 50-mL water sample from each tank on day 6 (based on the results of the first trial). The individual carp wet-weight was 15.5±3.0 g (n = 36, mean ±1 SD). The water samples were concentrated and extracted as described above.
3. We evaluated the effect of carp biomass/abundance on the eDNA concentration by placing 1, 5, or 10 fish in a tank (n = 4 tanks per treatment group). The water in the tanks was maintained at 19±1°C. On day 6, we collected a 50-mL water sample from each tank. The individual carp wet-weight was 15.8±2.8 g (n = 64, mean ±1 SD). The water samples were concentrated and extracted as described above.

### Experiment 2: pond experiment

To evaluate the relationship between carp biomass/abundance and eDNA concentrations in a more natural environment, we stocked fish into two artificial ponds in Dazaifu (33°31′N, 130°33′E), Fukuoka Prefecture. The experiment was conducted over a 21-days period between 30 November and 21 December, 2010. Our observational and field studies were permitted by the Center for Aquatic Environments Research, which is an owner of the ponds.

Ponds A and B had mean volumes of 54.5 and 41.1 m^3^ and average in/outflow rates of 2.9 and 2.8 L min^−1^, respectively. The water in the ponds was replaced at a rate of 7.7 or 9.8% per day, respectively. Conditions in the ponds were as follows: water temperature (pond A: 8.1–10.5, pond B: 8.3–10.8°C), conductivity (A: 0.016–0.017, B: 0.016–0.017 S m^−1^), dissolved oxygen (DO) (A: 9.4–10.0, B: 8.1–10.1 mg L^−1^), pH (A: 7.4–8.3, B: 7.6–8.1), turbidity (A: 5.0–14.0, B: 13.9–15.7 NTU), chlorophyll *a* concentration (A: 2.3–2.4, B: 1.8–2.8 µg L^−1^).

A single carp was held for one year in pond A prior to the experiment; pond B had been empty during this year. We then conducted preliminary eDNA analyses of the water in each pond and only found copies of common carp DNA in the water from pond A (day 0) (see Results). Following this, we weighed a single carp and placed it into pond B one week before the experiment began (n = 1 fish pond^−1^). On day 7, we collected 2-L water samples from the surface at three points in each pond. We then added to each pond an additional two fish that were fin clipped and weighed (n = 3 fish pond^−1^). On day 14, we collected water samples as described above, weighed and marked an additional 12 fish, and added six to each pond (n = 9 fish pond^−1^). On day 21, we collected water samples and captured the single, unmarked fish in pond A to measure its wet-weights. We used the mean value for the three sampling points per pond to represent the eDNA present on the day of sampling in each pond. The individual wet-weight of carp in both ponds was 982±575 g (n = 18, mean ±1 SD).

We concentrated eDNA by passing each water sample through either a 3.0-µm pore size filter (TSTP04700: Millipore) or a pre filter (12.0-µm pore size: TKTP04700) followed by a 0.8-µm pore size filter (ATTP04700). All three pore size filters were made of the same material (polycarbonate). Filter holders (Nalgene® NL300-4100) and a vacuum pump were used for the filtration. The filter discs containing the sample were transferred for storage to DNA-free 50-mL conical tubes (BD Falcon™) using tweezers. The tubes were completely sealed, immediately transported on ice in a cooling box to Kyoto Prefecture, and stored at −18°C until the following day. All filtration equipment was carefully rinsed with distilled water between filtration operations to prevent cross-contamination. To evaluate absence/presence of eDNA from common carp in both ponds, a preliminary qPCR assay was performed before the start of the experiment using the 0.8-µm filter.

The filter discs in each tube were soaked in 10-mL distilled water and stirred on a rotary shaker at maximum speed for 10 min. Then, the suspension was concentrated by centrifugal filtration (Amicon Ultra-15, 5000× g for 10–15 min). These procedures were repeated three times for each tube. We concluded that materials containing carp eDNA were sufficiently extracted from the filter discs because the pigmentation left by the filtration process had been adequately removed from the discs. The sample solutions were concentrated to volumes of 400 µL and stored in 1.5-mL microtubes (Eppendorf®) at −25°C. The eDNA from the sample solutions was extracted as described above.

### Field survey

We collected water samples by a boat from 21 sites at a freshwater lagoon (Iba-naiko) connected to Lake Biwa in Japan (35°11′N, 136°08′E) from 10:50–12:20 on 8 February, 2011. To prevent mixing of ambient water at each sampling site by movement of the boat, samples were carefully collected toward the upstream sites from the downstream sites. The lagoon was composed of five canal inflows and one canal outflow and had an area of 0.49 km^3^ and a maximum water depth of 3 m, though the majority of the lagoon was <1.5 m in depth. The lagoon was inhabited by a number of cyprinid fish species, including common carp, for breeding. The conditions in the lagoon were as follows: water temperature 5.9–10.7°C, conductivity 0.009–0.018 S m^−1^, DO 10.7–13.3 mg L^−1^, pH 7.2–7.8, turbidity 0.4–11.6 NTU, and chlorophyll *a* concentration 0.0–6.2 µg L^−1^. Water chemistry was monitored using a multi-water profiler (6600EDS; YSI, Yellow Springs, OH, USA). No permits were required for the field studies described here.

We collected 2-L water sample from the surface at each of 21 sites in the lagoon. The water samples were stored in DNA-free 4-L bottles and immediately transported to the laboratory. The 2-L water samples were filtered through a 3.0-µm membrane filter (142-mm diameter, C300A142C; Advantec, Saijo, Japan) using stainless steel filter holders (KS-142-US; Advantec). This pore-size filter was found to be the most suitable for concentrating water sample during the outdoor pond experiments (see Results). Each filter disc containing the sample was folded inward with tweezers and wrapped in DNA-free aluminum foil. The filter disc was immediately stored at −25°C until further analysis. All filtration equipment was carefully rinsed with distilled water between filtration operations to prevent cross-contamination.

To elute eDNA of the common carp, the filter discs containing the samples were placed in autoclaved 500-mL Nalgene® bottles using tweezers. The filter discs in the bottles were soaked in distilled water and stirred as described in pond experiment above. Then, the sample solution was concentrated and extracted as described above.

### DNA sequencing

To confirm the specificity of the primer set described above for the field samples, qPCR amplicons of all sites that were positive for the qPCR were directly sequenced after treatment with ExoSAP-IT (USB Corporation, Cleveland, OH, USA). Sequences were determined by a commercial sequencing service (Takara Bio, Tokyo, Japan).

### Statistical analyses

We evaluated the effect of temperature on the concentration of eDNA using one-way analysis of variance (ANOVA, α = 0.05). In the aquarium and pond experiments, we evaluated the relationship between eDNA concentration and biomass of carp per 1-L water sample using a Type II regression, and evaluated the relationship between the number of carp and biomass using a Type I regression. We used a general linear model (GLM, [18]) to evaluate the relationship between eDNA concentration and six environmental factors in the field survey: habitat type (shore or offshore), water temperature, DO, pH, conductivity, and chlorophyll *a* concentration. The factors in the GLM were standardized and centered. We selected the best GLM using a downward stepwise procedure based on the Akaike Information Criterion [19], [20]. Prior to the GLM analysis, we used a variance inflation factor (VIF) to check the collinearity of the factors. The maximum VIF of the GLM was 4.4, indicating that co-linearity among the factors did not significantly influence the results of the GLM. The eDNA concentration for ANOVA was log_10_ transformed to normalize the values based on the results of Shapiro-Wilk normality test (α = 0.05). ANOVA was performed using IBM SPSS Statistics (version 19, SPSS Japan Inc., Tokyo, Japan). The remaining statistical analyses were conducted in R ver. 2.13.0 [21].

## Results

### Experiment 1: aquarium experiments

**Figure 1:**
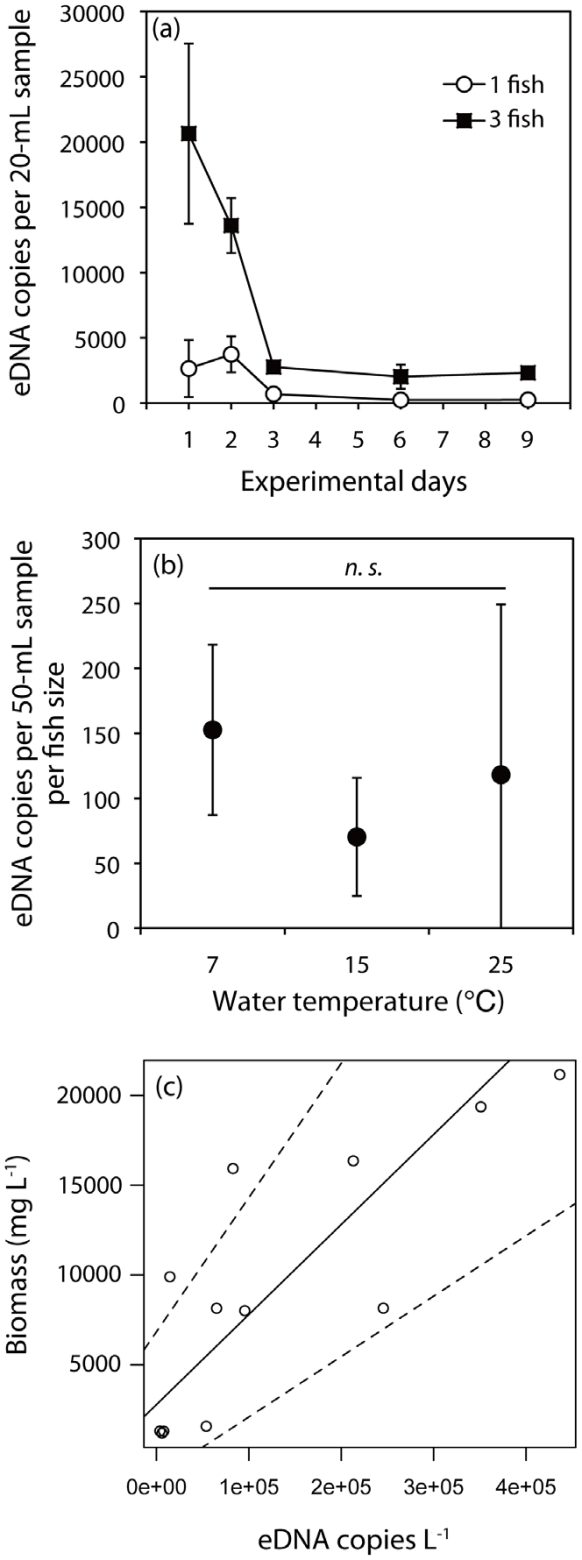
Relationships between the concentration of environmental DNA (eDNA) of common carp and three factors (duration, water temperature, and biomass) in aquarium experiments. (a) Time-dependent change in eDNA concentration at two biomass levels (one or three fish per tank). The error bars represent ±1 SD. (b) Effect of temperature on eDNA concentrations 6 d after introduction of fish to the tank. “*n.s.*" indicates no significant differences. The error bars represent ±1 SD. (c) Relationship between eDNA concentration and carp biomass per 1-L water 6 d after introduction of fish to the tank. The regression was significant (*p*<0.05). Dotted lines represent the lower or upper limits of the 95% confidence intervals for the slope of the regression.

The number of eDNA copies was highest in the tanks containing three fish on day 1 and in the tanks containing one fish on day 2 (3 fish: 20,647±6,909; 1 fish: 3,739±1,373 copies per 20-mL sample, mean ±1 SD) (Fig. 1a). The number of eDNA copies decreased gradually thereafter, with little change between days 6 and 9 (Range: 3 fish: 2,023–2,328, 1 fish: 235–246 copies). Thus, we concluded that the number of eDNA copies plateaued on day 6. Therefore, sampling for eDNA concentration in subsequent experiments was performed on day 6. No eDNA of carp were detected in the two negative control tanks without carp.

Water temperature had no effect on the number of eDNA copies (*F*_2,9_ = 1.31, *p* = 0.32; Fig. 1b). In contrast, there was a significant positive correlation between the number of eDNA copies and carp biomass per 1 L (*y* = 0.050*x*+2789, *R* ^2^ = 0.66, *p* = 0.001; Fig. 1c). Thus, eDNA can be used to estimate carp biomass using a Type II regression model. The correlation between the number of carp and biomass was significantly positive (*y* = 16866*x* – 625.20, *R* ^2^ = 0.96, *p*<0.001 in Supporting Information, Fig. S1a).

### Experiment 2: pond experiment

**Figure 2:**
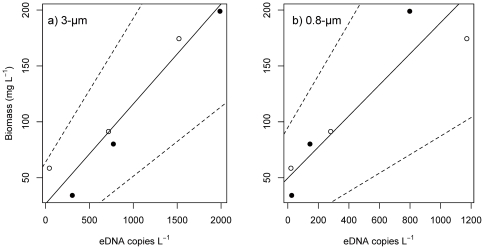
Relationships between the concentration of environmental DNA (eDNA) from common carp and their biomass in the outdoor pond experiment. The eDNA in the water samples was concentrated using (a) 3.0-µm and (b) 0.8-µm pore-sized filters. The regression line for both filter types showed that there was a positive relationship between eDNA concentration and carp biomass per 1-L water (see Results). Dotted lines represent the lower or upper limits of the 95% confidence intervals for the slope of the regression. The open and closed circles represent data from ponds A and B, respectively.

Before the start of the experiment (day 0), carp eDNA was detected in pond A (i.e. the pond with one fish for one year: 55 copies L^−1^ on the 0.8-µm filter) but not in pond B (i.e. the pond without fish prior to the experiment). During days 7–21, there was a positive relationship between the number of eDNA copies and carp biomass per 1 L for the samples processed using both 3.0-µm filters [pond A: 42±46 (1 fish pond^−1^), 717±254 (3 fish pond^−1^), 1,520±1,030 (9 fish pond^−1^); pond B: 305±167 (1), 773±561 (3), 1,988±1,371 (9), mean ±1 SD, n = 3] and 0.8-µm filters [pond A: 21±14 (1), 281±148 (3), 1,171±1,016 (9); pond B: 25±18 (1), 142±150 (3), 798±609 (9)]. The 3.0-µm filter yielded slightly better results when compared with the 0.8-µm filter (3.0-µm: *y* = 0.089*x*+26.57, *R* ^2^ = 0.93, *p* = 0.002; Fig. 2a) (0.8-µm: *y* = 0.139*x*+49.67, *R* ^2^ = 0.85, *p* = 0.009; Fig. 2b). The correlation between the number of carp and biomass was significantly positive (*y* = 7.937*x*+33.17, *R* ^2^ = 0.95, *p*<0.001 in Supporting Information, Fig. S1b).

### Field survey

**Figure 3:**
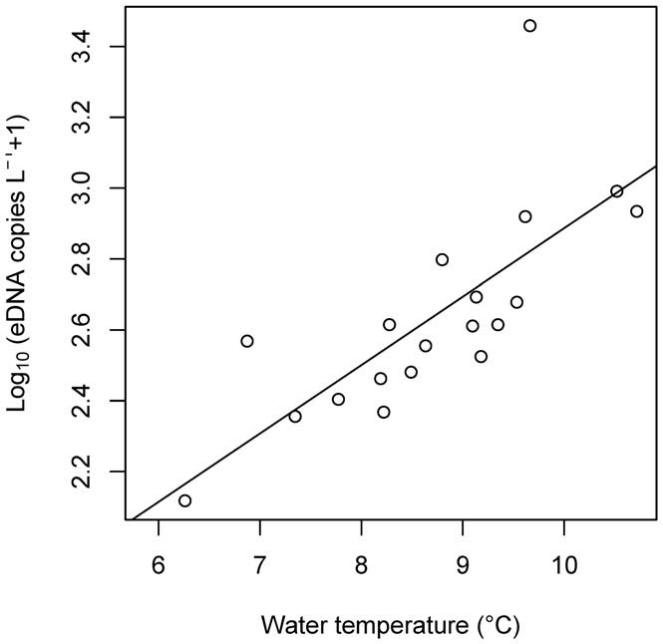
Relationships between concentration of environmental DNA (eDNA) from common carp and water temperature in Iba-naiko lagoon. The regression line showed a significant trend by GLM (*p*<0.05, see Results).

**Figure 4:**
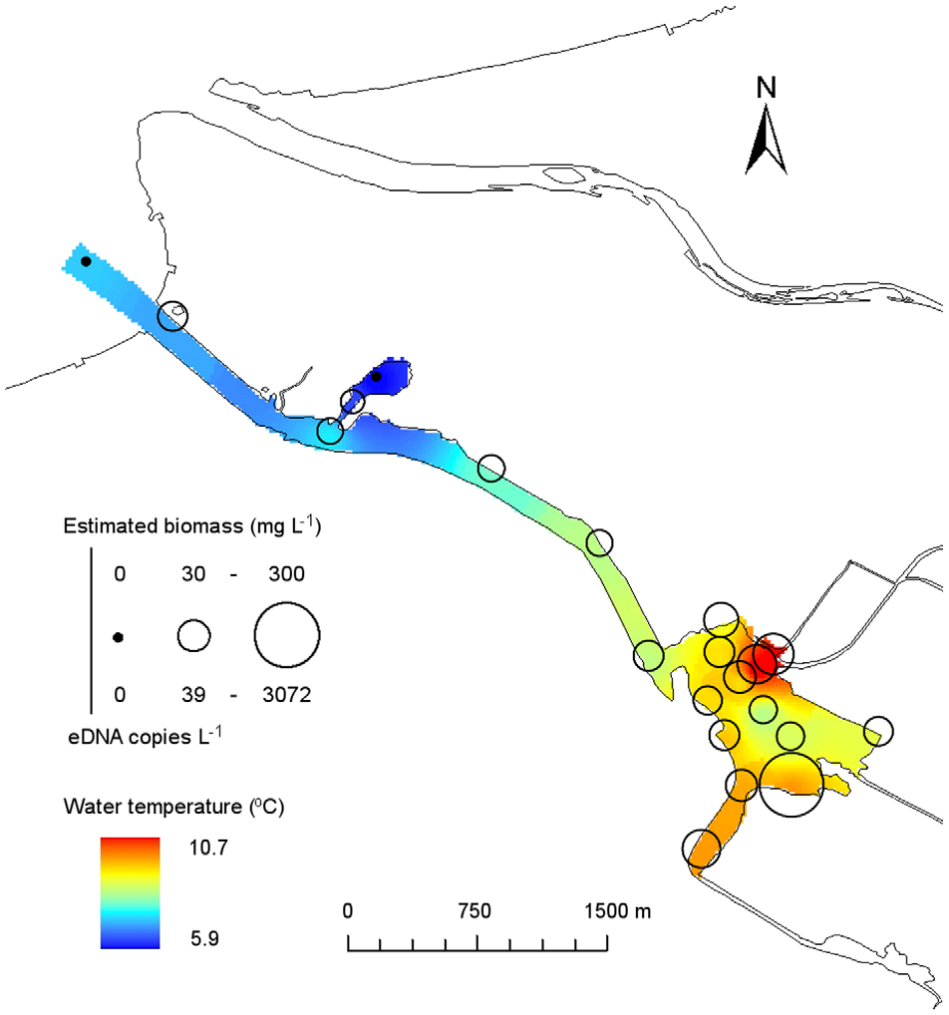
Estimates of carp biomass based on the concentration of environmental DNA (eDNA) in Iba-naiko lagoon, Japan. Estimated carp biomass (calculated from eDNA concentration) and water temperature at each site are represented by the circle size and color gradient, respectively.

The best GLM included water temperature for each site (Table 1). The relationship between the eDNA concentration of carp and water temperature was significantly positive (GLM, *p*<0.001; Fig. 3). The calibration for the 3.0-µm filter was applied to biomass estimation in the field survey, and the two sites that were negative for the qPCR assay were set to 0. The estimated carp biomass ranged from 0 to 282 mg L^−1^ (calculated from 0–2,875 copies L^−1^ in eDNA concentration) at the 21 sites in the lagoon. The estimated carp biomass and water temperature at each site is illustrated in Figure 4.

**Table 1:** Parameter coefficients and adjusted *R^2^* values of the full and best GLMs for the relationships between log_10_ (eDNA concentration+1) and six environmental factors.

	Full model	Best model
Habitat type (shore or offshore)	0.151	
Water temperature	**0.261**	**0.249**
Conductivity	−0.037	
Dissolved oxygen	−0.057	
pH of water	−0.052	
Chlorophyll *a* in water column	−0.100	
(Intercept)	**2.489**	**2.600**
Adjusted *R^2^*	0.449	**0.539**

The factors were standardized and centered. The best model was selected by a stepwise procedure using the Akaike Information Criterion. Bold values indicate a significant contribution (*p*<0.05).

To confirm the specificity of the primer set, we directly sequenced the qPCR amplicons, except for the two sites that were negative for the qPCR assay. All sequences from each qPCR amplicon at Iba-naiko lagoon were confirmed as being from common carp.

## Discussion

We developed a method for estimating the biomass/abundance of fish based on eDNA concentrations in water samples. Using this approach, we documented the distribution of eDNA concentration from a natural common carp population in a lagoon and assumed that it reflects the distribution of carp in natural habitats.

In the aquarium experiment, eDNA concentrations were highest immediately after the introduction of fish into the tanks. We speculate that the elevated concentrations resulted from increased discharge of DNA associated with increased activity of the fish during the acclimation period. After the first day or two, the eDNA concentration began to decline, and it had stabilized by six days. Dejean et al. [9] showed that eDNA concentrations decreased after the removal of the target animals. Our previous study demonstrated that the degradation rate of viral DNA (target length = 78 bp) was about 70% per day at 25°C [22]. Considering these facts, the initial decline in eDNA concentrations appear to have been due to the fish releasing less DNA as they acclimated to the tanks and their activities decreased in the first two days. By six days after their introduction to the aquaria, the release of eDNA from common carp appears to have reached an equilibrium with the rate of breakdown. Carp are thought to continuously release small quantities of eDNA. Moreover, the microbial community composition would be one of the important factors for the DNA degradation and accumulation. Therefore, future study is required to evaluate relationships between the microbial composition and eDNA concentration in natural environment.

In the both outdoor pond and aquaria experiments, we observed a highly positive correlation between the concentration of eDNA and carp biomass and a correlation between the number of carp and biomass. Thus, we speculated that eDNA concentrations in the environment reflected the biomass of the target species. This hypothesis is consistent with previous reports suggesting that eDNA could sometimes not be detected in a natural pond when the relative abundance of the target species was very low [3].

We were able to process large sample volumes and obtain sufficient eDNA using a 3.0-µm filter. Conversely, use of the 0.8-µm filters necessitated pre-filtration with a 12.0-µm filter to avoid clogging of the filter and was thus more labor-intensive. Previously, Goldberg et al. [5] used a 0.45-µm filter to concentrate 10-L water samples collected from stream and river systems. However, lentic environments such as lagoons and ponds contain significantly more suspended solids [23] that will clog filters with small pore sizes. We demonstrated that a 3.0-µm filter was suitable for collection of eDNA from a lentic environment.

In the freshwater lagoon, the eDNA concentration of common carp was highly variable among the 21 sites. Although water temperature had no effect on eDNA concentrations in our aquaria experiments, temperature appeared to be a major driver of carp eDNA distribution in the lagoon, with warmers areas having more eDNA. Water temperature has a significant effect on growth, metabolism, physiology, and immune function in fish [24]–[26]. Thus, fish species behaviorally select optimal temperatures and avoid areas that are suboptimal [27]. Our survey was conducted during the winter, when the water temperature was low (mean water temperature across sites: 8.5°C). To maintain their metabolism, common carp could prefer warmer water temperatures in winter. Indeed, carp appear to prefer warm habitats that do not exceed their optimal temperature [28]. Thus, eDNA concentration at each site may represent the temperature-dependent preferences of the carp. Moreover, there were two sites where carp eDNA was not detected, even though these two sites were only a few hundred meters from sites with eDNA. This result suggests that natural conditions (e.g., current and wind) cause little mixing of carp eDNA among nearby field sites and that carp eDNA is fully degraded at each site. Based on the significant correlation between water temperature and eDNA concentration by the statistical analysis, we thought that common carp was absent in the two sites where the water temperature was low. Consequently, the present study suggested that biomass data estimated from eDNA concentrations reflected the distribution of common carp in the natural environment. In addition to the six environmental factors analyzed in this study, the predation should be also important for the distribution of fish in the natural environment [29]. The modeling between the factors which are not considered in this study (i.e., predation) and the carp eDNA distribution in the field would enable better prediction of the distribution of common carp. In this study, we did not evaluate the effects of stressing environments, such as human activity and predation pressure, on the releasing rate of eDNA. Considering that stress could alter the fish behavior and consequently the release rate of DNA as observed in the beginning of the aquarium experiments, further study linking the stressing environments and eDNA concentration might be needed.

In summary, we developed an eDNA-based method to estimate the biomass of freshwater fish species. Using this method, the species biomass in natural environments can be estimated more easily and rapidly than using traditional methods, such as mark and recapture. Our method may be used to monitor seasonal changes in eDNA concentrations to predict important microhabitats for reproduction, feeding, and refuge of a target species. Fish biomass can be estimated using non-invasive water samples, and the data can be used to aid management plans for the conservation of populations, communities, and ecosystems. Moreover, documentation of the relationship between estimated host biomass and the concentration of pathogenic viruses in the natural environment might be used to predict the prevalence of infectious diseases (e.g. *Cyprinid herpesvirus 3* (CyHV-3) disease, [22]). To improve the accuracy of this method, future experiments should focus on collecting more field data and comparing this method with other estimation methods.

## Supporting Information

Figure S1 **Relationship between the number of carp and biomass for the aquarium experiment (a) and the outdoor pond experiment (b).** The regression was significant (*p*<0.05). In (b), the open and closed circles represent data from ponds A and B, respectively.
(TIFF)

Table S1 **Multiple alignment for all fish species in Lake Biwa.** We obtained all available cytochrome *b* sequences for all fish species known to habit in Lake Biwa. The consensus sequence for each fish species was determined by the EDNAFULL scoring matrix in eBioX software (http://www.ebioinformatics.org/index.html). The multiple alignment was performed using the Kalign algorithm in eBioX. Asterisks indicate non-target species that could potentially be amplified by our primer set (see Materials and Methods for details).
(XLS)
