# Supplementary figures and images for: Estimation of Fish Biomass Using Environmental DNA
Source: PLoS One. 2012 Apr 26;7(4):e35868. doi: 10.1371/journal.pone.0035868 (PMC3338542; doi:10.1371/journal.pone.0035868)

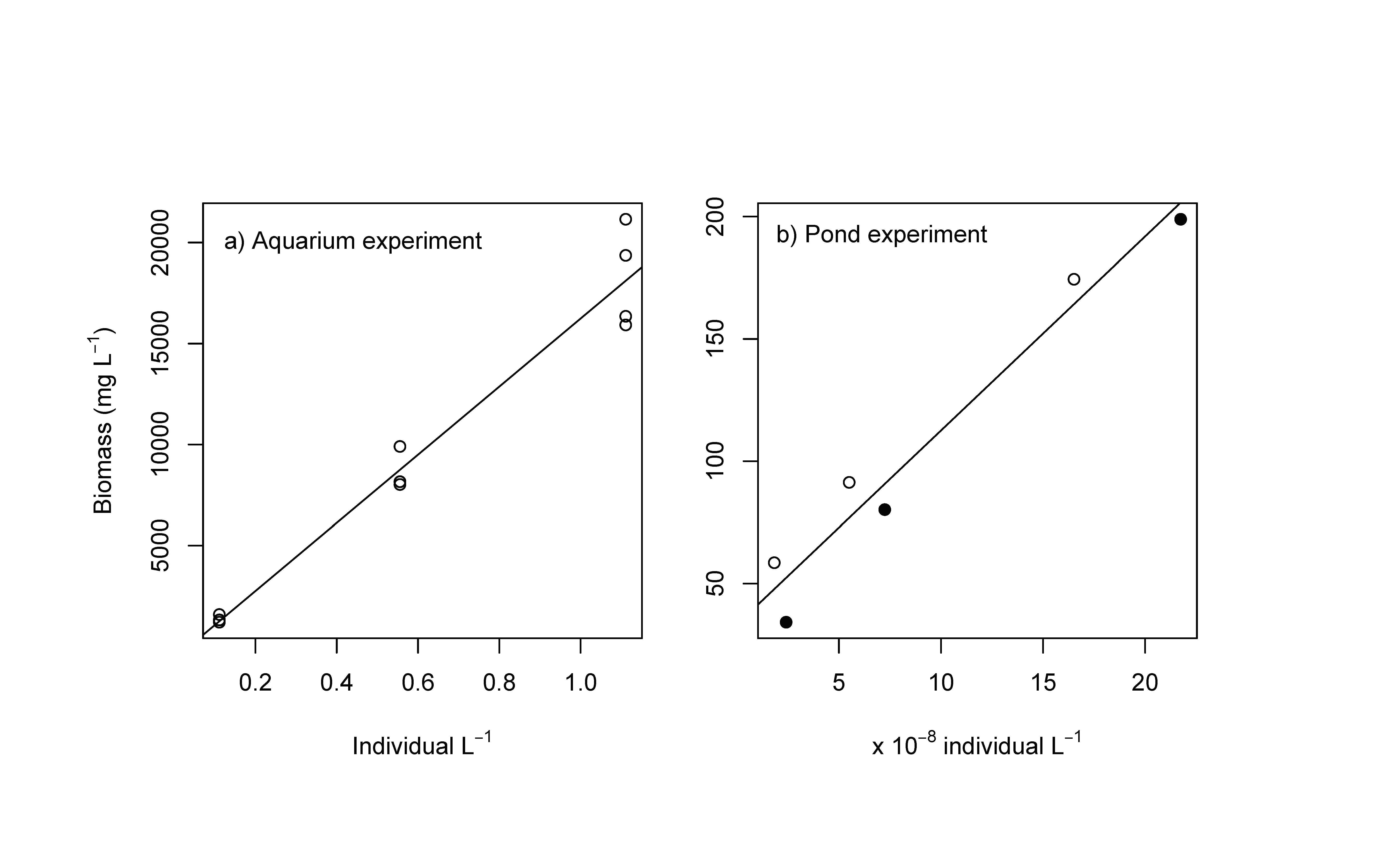

Supplement: Figure S1 — Relationship between the number of carp and biomass for the aquarium experiment (a) and the outdoor pond experiment (b). The regression was significant (p<0.05). In (b), the open and closed circles represent data from ponds A and B, respectively. (TIFF) [file pone.0035868.s001.tiff]
